# Modelling the impact of targeted anthelmintic treatment of cattle on dung fauna

**DOI:** 10.1016/j.etap.2017.07.012

**Published:** 2017-10

**Authors:** Andrew S. Cooke, Eric R. Morgan, Jennifer A.J. Dungait

**Affiliations:** a School of Veterinary Sciences, University of Bristol, Bristol Life Sciences Building, 24 Tyndall Avenue, Bristol, BS8 1TQ, United Kingdom; b Institute for Global Food Security, Queen’s University Belfast,University Road, Belfast, BT7 1NN, United Kingdom; c Sustainable Agriculture Sciences, Rothamsted Research, North Wyke, Okehampton, Devon, EX20 2SB, United Kingdom

**Keywords:** Anthelmintic resistance, Helminth, Antiparasitic, Targeted selective treatment, Refugia, Agriculture, Environment

## Abstract

The insecticidal properties of many anthelmintics pose a risk to dung fauna through the effects of drug residues in dung on the activity, oviposition and development of dung-dwelling invertebrates. Reductions in dung fauna numbers can inhibit dung degradation, which may impact biodiversity and nutrient cycling on farms. A simulation model was created to predict the impact of antiparasitic drugs on cattle dung fauna, and calibrated using published data on the dung-breeding fly *Scathophaga stercoraria*. This model was then tested under different effective dung drug concentrations (EC) and proportions of treated cattle (PT) to determine the impact under different application regimens. EC accounted for 12.9% of the observed variation in *S. stercoraria* population size, whilst PT accounted for 54.9%. The model outputs indicate that the tendency within veterinary medicine for targeted selective treatments (TST), in order to attenuate selection for drug resistance in parasite populations, will decrease the negative impacts of treatments on dung fauna populations by providing population refugia. This provides novel evidence for the benefits of TST regimens on local food webs, relative to whole-herd treatments. The model outputs were used to create a risk graph for stakeholders to use to estimate risk of anthelminthic toxicity to dung fauna.

## Highlights

• Assessment of the impact of anthelmintic targeted selected treatment on invertebrates.
• Practical risk matrix to assess the impact of treatment programs.
• Novel approach to life cycle modelling of invertebrates.
• Targeted selective treatment mitigates ecological impact of anthelmintics via refugia.

## 1 Introduction

Anthelmintic drugs are widely and routinely administered to grazing livestock to control gastrointestinal nematodes and other parasites. Anthelmintics are typically not fully metabolized within the host animal and residues of the drugs are often excreted in dung (McKellar et al., 1993) (and urine (McKellar, 1997)) and can therefore exert non-target effects on invertebrate fauna which spend part, or all, of their life cycle in dung (Floate, 1998a, Floate, 1998b, Gover and Strong, 1995, Madsen et al., 1990, Sommer et al., 1992, Sutton et al., 2014) and also on soil invertebrates (Scheffczyk et al., 2016). Such effects include inhibited motility, oviposition, emergence, and reduced dung pat colonisation (Floate, 1998a, Floate, 1998b, Gover and Strong, 1995, Suarez et al., 2003). Invertebrate dung fauna significantly contribute to the degradation of dung through physical processes and therefore reductions in the activity and populations of degradative fauna can to slow dung degradation (Madsen et al., 1990, Wall and Strong, 1987) with potential knock-on effects on important local processes., including local ecology (Beynon, 2012, Strong, 1993, Wall and Beynon, 2012) and epidemiology. In recent years, the mounting resistance of gastrointestinal parasites of domestic livestock to anthelmintic drugs has led to a shift away from whole-herd treatments, and recommendations for targeted selected treatment (TST) (Charlier et al., 2014) of only part of the herd. This strategy aims to generate refugia from drug exposure among parasite populations, slowing the development of resistance. In principle, refugia from drug residues ought also to be generated for dung fauna, supporting their populations; however, to date no systematic attempts have been made to evaluate this possibility.

The ability to assess and predict the impact of anthelmintics and other routine veterinary medicines on the wider environment is essential for informed drug development and policy in agriculture. In particular, parasite control practices that slow the development of resistance to commonly administered anthelmintics are essential to sustainable livestock production systems. However, the scale and complexity of the drug-dung-fauna system is challenging to observe and quantify *in vivo* and is difficult to fully represent under controlled laboratory conditions. Modelling techniques are the best alternatives to address these issues by allowing for the manipulation of a wide range of variables specific to individual field scenarios, and rapid assessments of the potential impacts of new parasite control and other management practices on dung fauna. Boxall et al. (2007) developed a screening index for assessing the impact of veterinary medicines on dung flies. The index was simple and allowed for estimates to be calculated with relatively small amounts of data, allowing for rapid screening of multiple drugs. The index assessed impact by multiplying three variables: proportion of cattle treated, proportion of time of faunal contact with dung, and dung toxicity. A central assumption was that the three variables are equally weighted, but this assumption inadvertently creates a potential mathematical ceiling to drug toxicity. Vale and Grant (2002) took a different approach in their development of a model to assess the impact of insecticide-contaminated dung on dung fauna. The model considered a broad and novel range of variables including the response to distinct adverse ecological events on insect life cycle stages and dung-insect interactions which aided the understanding of the importance of refugia for the ecology different species of invertebrates.

Here, we test the hypothesis that the proportion of cattle treated (PT) with anthelmintics has a greater influence on *Scathophaga stercoraria* populations than the strength of drug residue in dung (EC). We build on previous theoretical and modelling approaches to create a new modelling approach to simulate the drug-dung-fauna system and evaluate the potential impacts of antiparasitic drug use in grazed cattle production systems. We use the model to consider how varying treatment regimens administered by veterinarians for the purpose of livestock health and welfare have non-target influences on dung invertebrates, and to provide a risk graph to inform stakeholders in sustainable livestock production systems.

## 2 Methods

### 2.1 Model description

A simulation model was created using NetLogo 5.0.4 (Wilensky, 1999) to estimate the impact of a hypothetical anthelmintic that expressed insecticidal properties when excreted in dung by cattle in a grazed field, upon a model dung invertebrate. A 2-dimensional virtual pasture system was created, occupied by a herd of cattle and a population of the model invertebrate. All actions and interactions presented were simulated hourly time-steps for each individual cattle or invertebrate, as appropriate.

### 2.2 Model components

The model simulated the interaction between a model dung invertebrate and cattle defecation behaviour, and the potential for invertebrate survival to be changed by different concentrations of anthelmintic residues in the dung.

The model invertebrate was the yellow dung fly *Scathophaga stercoraria*. The model utilized published data (Table 1) to simulate the life cycle of *S. stercoraria* in a temperate cattle grazing system. *Scathophaga stercoraria* is a well-studied dung fauna species, for which detailed information on life cycle parameters is widely available. The species is highly abundant across the northern hemisphere, and some of its life cycle stages are dependent on dung.

**Table 1:** Model variables and values used for simulations. Mean values are fixed constants other than those with a standard deviation (S.D.) which were random variables within a standard normal distribution generated by random number generator using NetLogo 5.0.4. Sources: ^1.^Blanckenhorn, (1997), ^2.^Blanckenhorn et al. (2010), ^3.^Römbke et al. (2009), ^4.^Martin et al. (2004), ^5.^Aland et al. (2002), ^6.^Gary et al. (1970), ^7.^Oudshoorn et al. (2008), ^8.^Sahara et al. (1990), ^9.^Villettaz Robichaud et al. (2011), ^10.^ Floate (1998), ^11.^Vale and Grant (2002), ^12.^Geiger (2010), ^13.^Parker (1970).

Variable^source^	Value
Dung fauna (*S. stercoraria*)	
Adult life span (emergence to death)^1^	44 days
Juvenile period (egg to emergence)^2^	22 days
Female:male ratio^1^	1:1
Dung preference^3^	0
Progeny to reach adulthood^4^	10.8 (2.9)

Cattle and dung	
Mean daily defecation rate (pats per day)^5–9^	11.2 (2.4)
Dung attractive period (with drug residue) to *S. stercoraria*^3,10,11^	5 days
Dung attractive period (no drug residue) to *S. stercoraria*^3,10,11^	5 days
Mean dung pat carrying capacity for juveniles^12^	4.3
Season length^13^	6 months
Number of cattle	20

The model cattle were based on published data on temperate grazing commercial beef and dairy herds (Table 1). There were two components to cattle behaviour: (1) defaecation frequency, and (2) randomized movement across a field. The cattle were treated or untreated with a hypothetical anthelmintic, producing toxic or non-toxic dung, respectively. The proportion of cattle treated (PT) ranged from 0 to 1 in increments of 0.1 and was specific as an independent variable in each simulation,

The rate of defecation of model dung by the model cattle and its mean carrying capacity for *S. stercoraria* was based on published data for temperate commercial beef and dairy systems (Table 1). The model dung were toxic or non-toxic. The strength of the toxicity, i.e. effective concentration (EC) ranged from 0 to 1 in increments of 0.1 and was specific as an independent variable in each simulation. The dung became unattractive for *S. stercoraria* regardless of toxicity after a simulated 120 h.

A starting population of 100 individuals of *S. stercoraria*, covering a random distribution of ages within typical life expectancy for *S. stercoraria*, were simultaneously introduced to the system. They actively sought out cattle dung in order to produce off-spring with no preference for toxic or non-toxic dung. Population fitness responses of the *S. stercoraria* to contact with toxic dung was based on the interaction between PT and the specific EC.

Primary assumptions were:

(i) the model dung toxicity retained a constant toxicity for 120 h
(ii) there were no sub-lethal effects of the anthelmintics upon *S. stercoraria*
(iii) there were no other sources of mortality exist for *S. stercoraria* other than toxicosis or exceedance of life span
(iv) the population of *S. stercoraria* is isolated.

No values or weightings of variables within the model were assumed or given arbitrary values.

### 2.3 Application of modeling approach

The model was run 605 times. Each run simulated 4380 h (6 months) using all combinations of 11 PT values and 11 EC values, totaling 121 unique sets of parameter values. There were five repeats of each set, with variable outcomes depending on values simulated from normal distributions: the mean of each set of repeats was used for statistical analyses. The Anderson-Darling normality test was conducted on residuals for the dependent variable of final population size at the end of the simulated period to ensure appropriateness for parametric testing. This was followed by Pearson’s correlation analyses of final population size versus PT and EC. Multiple regression analyses were then conducted to attribute how much of the variation in final population size was due to PT and EC, respectively.

A number of individual paired simulations were run to evaluate the index created by Boxall et al. (2007). These simulations were performed in pairs in which the product of PT and EC were equal, but the individual values of PT and EC in each pair were not equal. To achieve this the values for PT and EC of pair 1 were switched to form pair 2 (Table 2). For the Boxall et al. (2007) model to agree with the presented model, there should be no significant different between pairs that meet the aforementioned assumptions. Final population numbers from simulations were then subject to the Paired *T*-test.

**Table 2:** Values of PT and EC for paired simulations in order to evaluate Boxall et al. (2007) model.

	Group A		Group B	
Pair no.	EC	PT	EC	PT
1	0.0	1.0	1.0	0.0
2	0.1	0.9	0.9	0.1
3	0.2	0.8	0.8	0.2
4	0.3	0.7	0.7	0.3
5	0.4	0.6	0.6	0.4

## 3 Results

**Fig. 1:**
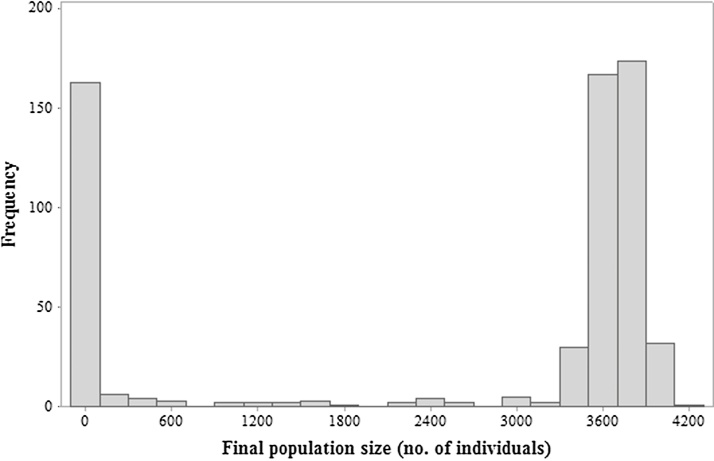
Distribution of final population sizes of *S. stercoraria* from all (605) simulations of PT and EC pairings.

**Fig. 2:**
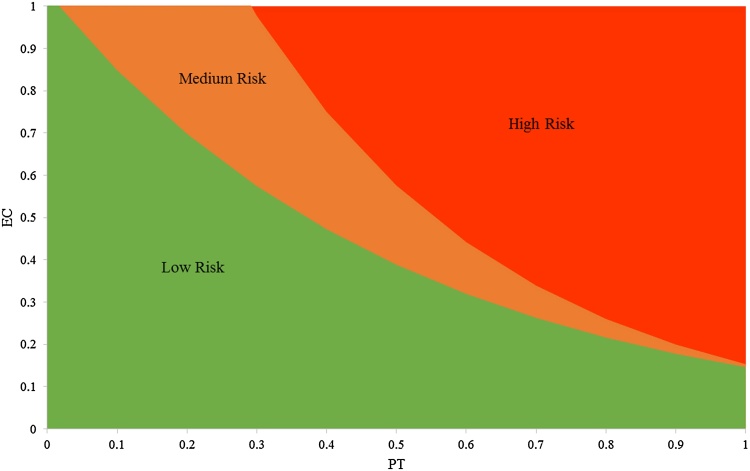
Risk thresholds for the impact of anthelmintics on *S. stercoraria*. “Low Risk” refers to PT (proportion of treated cattle) and EC (effective concentration) combinations that result in final populations exceeding Q1 of the maximum fitness group, “Medium Risk” to those falling between Q0 and Q1 and “High Risk” to those falling below Q0.

The distribution of final population sizes across all simulations was non-normal (Anderson-Darling, *p* *=* < 0.005). The data shows two distinct groupings based on final population size, one at 0 and the other in the region of 3100–4300 (Fig. 1). This latter group, the ‘maximum fitness’ group, had a normal distribution (*p* = 0.383). Quartiles for the maximum fitness group were measured as Q0 = 3259, Q1 = 3597, Q2 = 3703, Q3 = 3798, Q4 = 4197. PT and EC combinations that resulted in final populations of <Q0, and therefore outside of this group, were considered as high risk. Combinations that fell between Q0 and Q1 were considered medium risk, and all over combinations resulting in final populations >Q1 were considered low risk (Fig. 2).

In general, incremental increases in PT and EC at low levels had little effect on final population size (no. of individuals), but a tipping point was reached beyond which the population decreased exponentially (Table 3). Rising EC values from 0.0 to 0.5 brought about gradual decreases in final population size; however, as EC exceeded 0.5 its effect on population size reduced. In contrast, rising PT values of 0.0–0.5 had little impact upon population sizes, but as PT exceeded 0.5 there was a rapid drop in population size.

**Table 3:** Mean simulated final population size for varying proportions of cattle treated (PT) and effective dung drug concentrations (EC). PT and EC range from 0 to 1.0 in intervals of 0.1, so simulations were conducted for 121 scenarios, representing every PT and EC value combination.

		PT										
		0.0	0.1	0.2	0.3	0.4	0.5	0.6	0.7	0.8	0.9	1
EC	**0.0**	3678	3676	3701	3763	3645	3739	3770	3696	3590	3642	3608
	**0.1**	3675	3681	3734	3699	3652	3765	3767	3650	3574	3598	3713
	**0.2**	3764	3697	3692	3685	3659	3629	3741	3635	3044	1353	338
	**0.3**	3789	3753	3655	3777	3659	3777	3409	2950	1422	0	0
	**0.4**	3859	3721	3758	3722	3603	3789	2784	1756	0	0	0
	**0.5**	3667	3620	3705	3807	3294	3325	2112	0	3	0	0
	**0.6**	3658	3661	3701	3777	3783	3147	1481	85	0	0	0
	**0.7**	3738	3786	3816	3723	3755	2902	1385	762	0	0	0
	**0.8**	3750	3661	3671	3655	3660	3310	1634	744	4	0	0
	**0.9**	3761	3665	3689	3724	3178	2594	1586	8	0	0	0
	**1**	3790	3680	3754	3564	3745	2337	0	724	0	0	0

The residuals of the complete data for all experimental runs were normally distributed (as tested by Anderson-Darling test, *p* = 0.281) and thus no transformation was required for parametric analyses. A Pearson’s correlation analysis showed that final population size was significantly correlated with PT (−0.694, *p* *=* < 0.001) and EC (−0.336, *p* = <0.001). A subsequent multiple regression calculated the total variance of final population explained by PT and EC together, R^2^, to be 67.8% (*p* < 0.001). Further individual regressions showed that PT explained 54.9% (p = < 0.001) and EC explained 12.9% (p = < 0.001) of total variance in final population size.

The paired *t*-tests, for the purpose of evaluating Boxall et al., showed a statistically significant intra-pair difference (*t* = 2.43, *p* = 0.023) and therefore the H_0_ was rejected in favour of the H_a_, that there is an intra-pair difference. That is: simulations of which the sum of PT and EC are equal do not yield equal results.

## 4 Discussion

In this study, we used a novel simulation to test the hypothesis that PT had a greater impact on the population size of *S. stercoraria* than EC. The outcomes of 605 simulations of 121 parings of PT and EC indeed indicate that this hypothesis can be accepted. The distribution of data predicted that populations of *S. stercoraria* were generally resilient and can maintain stable numbers up until a tipping point at which mortality becomes probable. As such, our model develops the concept of the screening level index (Boxall et al., 2007) through simulation modelling using published data about key life cycle parameters that could strongly influence drug-insect interactions. We propose that this new approach provides a better justified mechanistic framework for impact assessment, which will improve recommendations of use of veterinary medicines with consideration for livestock dung ecology and wider impacts on the environment.

Cow pats in grazed systems without drug residues may provide an important reservoir of biodiversity, allowing maintenance populations of coprophagic fauna that are important for ecosystem services including nutrient cycling, carbon cycling and soil quality, e.g. dung beetles and insect larvae. Therefore, TST, as opposed to a whole-herd treatment, is recommended to reduce the impacts of drug treatment on local ecosystems, with additional economic benefits through reduced inputs on-farm (Charlier et al., 2012), ecosystem service delivery, and ensuring sustainable parasite control options in the longer term through slowing of anthelmintic resistance. However, in our model, *S. stercoraria* populations were assumed to be isolated, but wider consideration of the spatial variation in the local food web including the availability of drug-free dung in the wider environment would more closely represent the complexity of farming systems.

Our model provides a framework that is adaptable to dung-breeding insect species other than *S. stercoraria*. Its application to other target species, however, would require further empirical information on the toxicity of various drugs, as faecal residues, on specific fauna. Moreover, life cycle parameters specific to other species would be required, although the model could also be used to explore parameter space and identify broad characteristics of species that are likely to be vulnerable to anthelmintic residues in dung, and the extent to which these might be attenuated by TST. Since the model framework was developed using a bottom-up approach, it lends itself to constructive adaptation and expansion. With sufficient observational data there is scope for future models, within such a framework, to increase in complexity and realism. Expansion of the model to represent multiple invertebrates at farm level would enable holistic landscape-scale impact assessments and attenuation strategies.

The use of veterinary medicines, with non-target insecticidal properties, is ubiquitous and therefore the applicability of observed results may be equally wide, and this model framework can be adapted to any system, anywhere, given workable parameter estimates. The data from the literature that provided the foundation for the model was predominantly derived from studies in temperate regions, and we recognize that climatic differences may have a significant impact on the ecotoxicity of such medicines (Kryger et al., 2005). Moreover, the model was based upon a set-stocked system but could be modified to represent more extensive systems, including ranch/range rearing of cattle in the USA, South America, and Australia. The model provides a framework for the development of future similar work and could be applied to scenario-testing using the specific characteristics of different cattle production systems across the world.

Despite the high profile, global threat of drug resistance, the long-term impacts of drugs, especially antiparasitics with non-target insecticidal properties, are largely unknown. The topic is a key area for future work to enable effective assessment and regulation of the use of veterinary medicines, with regards to their impact on all aspects of biodiversity (Adler et al., 2016). Future work should also include economic analysis, in order to balance short-term production gains with longer term environmental impacts. There is likely to be a utilitarian argument to use veterinary medicines in a more sustainable manner, including the utilization of preventative and non-pharmaceutical methods (Kaplan and Vidyashankar, 2012, Papadopoulos, 2008, Wolstenholme et al., 2004). The emergence of part-herd anti-parasitic treatments, or TST, is an example of a more efficiently targeted approach to chemical utilization in agricultural systems, which has potential long-term economic benefits, as well as reduced environmental impacts. The current model shows this synergy in quantitative terms for a model insect species, and provides a framework for impact assessment and optimization of TST strategies across a wider range of dung fauna, including those of conservation relevance.

## Author contributions

All authors contributed to drafts of the paper. EM devised the study and advised on model assumptions and parameter values. AC compiled and ran the model, analyzed results and led the writing of the paper. JD supervised AC and provided editorial support.
